# Chitosan-coated nanostructured lipid carriers for effective brain delivery of Tanshinone IIA in Parkinson’s disease: interplay between nuclear factor-kappa β and cathepsin B

**DOI:** 10.1007/s13346-023-01407-7

**Published:** 2023-08-19

**Authors:** Donia M. Hassan, Amal H. El-Kamel, Eman A. Allam, Basant A. Bakr, Asmaa A. Ashour

**Affiliations:** 1 Department of Pharmaceutics, Faculty of Pharmacy, Alexandria University, 1 Khartoum Square, Azarita, Alexandria Egypt; 2 Department of Medical Physiology, Faculty of Medicine, Alexandria University, Alexandria, Egypt; 3 Department of Zoology, Faculty of Science, Alexandria University, Alexandria, Egypt

**Keywords:** Phytomedicines, Neurodegenerative disorder, Cathepsin B, Tyrosine hydroxylase, Chitosan

## Abstract

Parkinson’s disease (PD) is the second most common progressive neurodegenerative disorder associated with increased oxidative stress, the underlying vital process contributing to cell death. Tanshinone IIA (TAN) is a phytomedicine with a documented activity in treating many CNS disorders, particularly PD owing to its unique anti-inflammatory and antioxidant effect. However, its clinical utility is limited by its poor aqueous solubility, short half-life, and hence low concentration reaching targeted cells. This work aimed to develop a biocompatible chitosan-coated nanostructured lipid carriers (CS-NLCs) for effective brain delivery of TAN for PD management. The proposed nanosystem was successfully prepared using a simple melt-emulsification ultra-sonication method, optimized and characterized both in vitro and in vivo in a rotenone-induced PD rat model. The developed TAN-loaded CS-NLCs (CS-TAN-NLCs) showed good colloidal properties (size ≤ 200 nm, PDI ≤ 0.2, and ζ-potential + 20 mV) and high drug entrapment efficiency (> 97%) with sustained release profile for 24 h. Following intranasal administration, CS-TAN-NLCs succeeded to achieve a remarkable antiparkinsonian and antidepressant effect in diseased animals compared to both the uncoated TAN-NLCs and free TAN suspension as evidenced by the conducted behavioral tests and improved histopathological findings. Furthermore, biochemical evaluation of oxidative stress along with inflammatory markers, nuclear factor-kabba β (NF-Kβ) and cathepsin B further confirmed the potential of the CS-TAN-NLCs in enhancing brain delivery and hence the therapeutic effect of TAN of treatment of PD. Accordingly, CS-TAN-NLCs could be addressed as a promising nano-platform for the effective management of PD.

**Graphical abstract:**

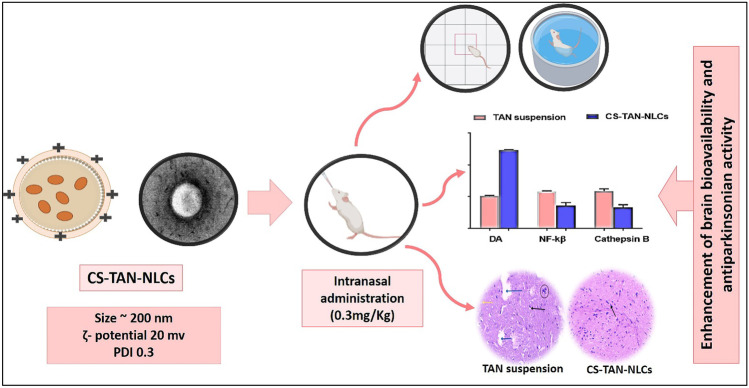

## Introduction

Parkinson’s disease (PD) is regarded as the second most common neurodegenerative disorder after Alzheimer’s disease. Among its prominent hallmarks is the loss of dopaminergic neurons in substantia nigra pars compacta (SNpc) with abnormal accumulation of α-synuclein in the form of Lewy bodies intracellularly [1]. Decrease in motor function and non-motor impairments (depression and anxiety) are considered the key symptoms of PD [2]. The possible underlying causes for these manifestations are mitochondrial dysfunction, oxidative stress, protein aggregation, impaired autophagy, and neuroinflammation [3].

Up till now, no perfect treatment exists for management of PD and therapy is mainly focusing on the relief of symptoms [4]. The current treatment includes the use of dopamine agonists (levodopa) and monoamine oxidase (MAO) inhibitors. Nevertheless, as the disease worsens, the efficacy of the conventional therapy decreases producing end-of-dose adverse effects called wear-off symptoms characterized by the recurrence of both motor and non-motor manifestations [4]. Recently, the focus on discovering phytomedicines has been growing considerably as a method of finding safer alternatives to synthetic drugs. Among them, Tanshinone IIA (TAN), a lipophilic diterpene extracted from the roots of a Chinese herb called *Salvia miltiorrhiza*, was reported to possess diverse biological activities against many human diseases involving neurological disorders, particularly PD [5]. Several mechanistic studies were conducted in experimental models to elucidate its antiparkinsonian efficacy through prevention of dopaminergic neuronal loss via anti-apoptotic, anti-inflammatory, and antioxidant mechanisms [5].

Moreover, TAN could inhibit the expression of NADPH oxidase and iNOS in substantia nigra and hence preventing nigrostriatal dopaminergic neuronal degeneration [6]. It can also regulate the DJ-1 and Nrf2/HO-1 signaling pathways [7]. Other studies investigated its neuroprotective effects in PD mostly mediated via suppressing oligomerization and fibrillation of α-synuclein, reducing oxidative stress and upregulating the expression of tyrosine hydroxylase in substantia nigra [5].

Despite its great pharmacological activity, its clinical utility is hampered by being among class IV drugs in BCS having both low aqueous solubility and permeability [8]. Furthermore, it suffers from both first-pass intestinal and hepatic metabolism as well as exposure to P-glycoprotein efflux and hence has short half-life in vivo [8]. Consequently, numerous attempts were conducted aiming to enhance its bioavailability via its incorporation into various nanocarriers, such as liposomes [9], nano-emulsions [10, 11], lipid nanocapsules [8, 12], solid lipid nanoparticles [13, 14], and nanostructured lipid carriers [15].

Incorporation of TAN in a suitable nanosystem, administered through intranasal route, could achieve an effective drug delivery to brain in management of PD by circumventing the blood brain barrier. Following intranasal administration, the medication reached the brain directly through the olfactory pathway and hence preventing drug delivery to non-target sites [16].

Based on the hydrophobic nature of the drug, NLCs offer a tailored design capable of tackling the enormous TAN delivery problems. Several advantages have been demonstrated by NLCs, such as avoiding the use of toxic organic solvents during preparation, allowing for controlled drug release, biocompatibility, and protecting the loaded medications from pre-systemic metabolism [17].

Surface modification of NLCs with chitosan coating could be of great value in management of PD since it could extend their residence time in the nasal cavity after their intranasal administration owing to its mucoadhesive and penetration enhancement properties [1, 18]. The enhanced transport of CS-coated NLCs across in vitro olfactory cell monolayers was proven compared to the uncoated formula [18]. Furthermore, low molecular weight chitosan was reported to have neuroprotective and antioxidant properties [19, 20].

Although numerous researches were reported in literature concerning the use of various TAN nanocarriers in the management of several diseases, as far as we know, none of them addressed their utility in treatment of PD.

In this regard, the present study aimed to develop CS-TAN-NLCs as an effective nanoformulation for PD therapy after intranasal administration. Following optimization, the elaborated nanosystem was subjected to full in vitro characterization. As well, a rat model with rotenone-induced PD was used to test its in vivo efficacy. Improvement of both motor and non-motor PD symptoms was assessed by various behavioral tests, biochemical evaluation, and histopathological and immunohistological examinations. As far as we know, this study is the first to investigate the therapeutic potential of TAN loaded in a nanocarrier (CS-TAN-NLCs) in treatment of PD, to evaluate its antidepressant effect as well as to explore a new target for the neuro anti-inflammatory effect of TAN via inhibition of Cathepsin B.

## Materials and methods

### Materials

Tanshinone IIA (purity 98%) was purchased from Baoji Guokang Bio-Technology Co., Ltd, China. Labrafac™ lipophile WL 1349, Labrafac™ PG, Peceol™, Compritol^®^ 888 ATO, Precirol^®^ ATO 5, and Glyceryl monostearate were kindly gifted by Gattefossé S.A., Saint-Priest, France. Kolliphor^®^ HS15 and low molecular weight chitosan were obtained from Sigma-Aldrich, USA. Tween^®^ 80 and absolute ethanol were purchased from ADWIC, El-Nasr Pharmaceutical Chemicals Co., Cairo, Egypt. All other chemicals and organic solvents were of analytical grade.

### Screening of different liquid and solid lipids

The saturation solubility of TAN in various liquid lipids (Labrafac™ lipophile WL 1349, Labrafac™ PG, and Peceol™) was assessed by the addition of an excess amount of the drug to 1 g of each oil in small, stoppered vials. The obtained mixtures were vortexed for 5 min and then continuously stirred in a shaking water bath (type 3047; Kottermann, Hanigsen, Germany) at 100 rpm for 24 h at 25 °C. After another 24 h equilibrium, samples were centrifuged at 10,000 rpm for 10 min. Finally, the concentration of dissolved TAN in the filtered supernatants was determined by UV–vis spectrophotometry (Agilent Cary-60, USA) at 270 nm.

Regarding screening of the solid lipids, an amount of 1 g of each lipid (Compritol, Precirol, and Glyceryl monostearate) was heated 5–10 °C above its melting point. Increasing amounts of TAN were added until saturation was reached which is assessed visually [21].

### Preparation of blank NLCs

Blank NLCs were prepared by the simple emulsification sonication method described previously with some modifications [22]. In brief, the selected solid lipid (Compritol) was melted by heating at 75 °C followed by the addition of the appropriate liquid lipid (Labrafac™ lipophile) in a ratio of 3:2 w/w, respectively. The aqueous phase containing a mixture of emulsifiers Kolliphor^®^ HS15 and Tween^®^ 80 in a ratio 3:2 w/w, respectively, was heated to the same temperature and poured into the lipid phase. The mixture was magnetically stirred at 800 rpm for 15 min to form a coarse emulsion. To obtain a nano-dispersion, sonication for 15 min at 60 °C was performed using a probe sonicator (Bandelin Sonopuls, Germany) at 60% amplitude in pulsatile mode (2 s on and off). Finally, the prepared NLCs were left to cool slowly to 20 °C before being stored at 4 °C.

### Preparation of TAN-NLCs and CS-TAN-NLCs

For preparation of TAN-NLCs, the required amount of TAN was weighed and dissolved in the lipid phase and the same procedures implied for preparation of blank NLCs were applied.

As for CS-TAN-NLCs, they were prepared by simple titration method as previously reported [18]. In brief, 1 ml of the developed TAN-NLCs was added to different volumes of chitosan solution in 1% v/v acetic acid with different concentrations (0.1%, 0.2%, and 0.5% w/v). The mixture was kept under mild magnetic stirring (500 rpm) at 37 °C for 1 h. Effective chitosan coating was confirmed by size and ζ-potential measurements [23].

### Physicochemical characterization

#### Measurement of particle size and ξ-potential

The mean particle size and polydispersity index (PDI) of the different prepared NLCs were determined by Zetasizer (Malvern Zetasizer Nano-ZS, Malvern Instruments, Malvern, UK). Prior to measurements, samples were appropriately diluted with distilled deionized water and subjected to sonication for 5 min. Results were recorded as the average size, PDI, and ζ-potential measurements for three different samples.

#### Determination of TAN entrapment efficiency (EE)

For determination of TAN EE %, samples of different NLCs formulations were properly diluted with water and then subjected to centrifugation using Vivaspin^®^ 6 centrifugal ultrafilters (MWCO = 100,000, Sartorius, USA) at 6000 rpm for 30 min at 4 °C. The concentration of unentrapped TAN in the filtrate was quantified spectrophotometrically at 270 nm [8]. EE % was calculated using the following equation:

$$\mathrm{\% }EE= \frac{Total\;drug\;amount \left(mg\right)-amount\;of\;unencapsulated\;drug\;(mg)}{Total\;drug\;amount\;(mg)} \times 100$$

#### Morphological examination using transmission electron microscopy (TEM)

The morphology of the prepared TAN-NLCs and CS-TAN-NLCs was examined by TEM (Jeol, JEM-100 CX electron microscope, Tokyo, Japan). The nano-dispersions were properly diluted with filtered distilled deionized water. Samples were dropped onto copper grids, stained with uranyl acetate, and left for few min to dry out before examination.

#### Powder X-ray diffractometry (XRD)

To investigate the crystallinity of the TAN loaded in the prepared NLCs, XRD analysis was conducted on the dried samples using an X-ray diffractometer (X'Pert PRO diffractometer, Netherlands). For drying, liquid NLCs were mixed with Aerosil^®^ 200 in a ratio 4:1, respectively, and then left overnight in a desiccator. The diffraction pattern was performed in a step scan model with a voltage of 30 kV and a current of 30 mA. The scanning region of the diffraction angle, 2θ, was from 0 to 100. The tested samples were Aerosil^®^ 200, free TAN, and TAN-NLCs [8].

#### In vitro drug release

The release of TAN from different formulations (TAN-NLCs and CS-TAN-NLCs) as well as TAN suspension (in ethanol–water, ratio: 5.6:1, respectively) was conducted using the dialysis technique [8]. Briefly, an aliquot of 2 ml from each sample (containing 1 mg TAN) was put in pre-soaked dialysis bags (Visking^®^ 36/32, 24 mm, MWCO 12,000–14,000, Serva, USA). After that, the bags were placed in 75 ml of 50% v/v ethanolic water. The release experiment was carried out at 37 °C and 100 rpm in a thermostatically controlled shaking water bath. At predetermined time intervals (1, 2, 3, 5, 7, and 24 h), 2 ml of release medium was withdrawn and compensated with an isovolumic fresh medium. TAN concentration in the release medium was assayed spectrophotometrically at 270 nm. The study was performed in triplicate and results were presented as mean ± SD.

Drug release kinetics from different formulations were evaluated using model-dependent methods measured by the Excel add-in, DDsolver [24].

#### Shelf stability study

The shelf stability for the elaborated TAN-NLCs and CS-TAN-NLCs was assessed after their storage at 4 °C for 3 months. Samples were tested for any change in their colloidal properties as well as EE% every month throughout the study period.

### In vivo evaluation of the antiparkinsonian efficacy

#### Experimental animals

A total number of 35 adult male Wistar rats (180–220 g, 8 weeks old) were used. Animals were maintained in standard metal cages at 25 ± 1 °C and 65% relative humidity with a 12-h light/ dark cycle and free access to food and water. The experiment was carried out in accordance with protocol approved by the Animal Care & Use Committee (ACUC) of Faculty of Pharmacy, Alexandria University (Approval No: 062020128187).

#### Induction of PD

**Fig. 1:**
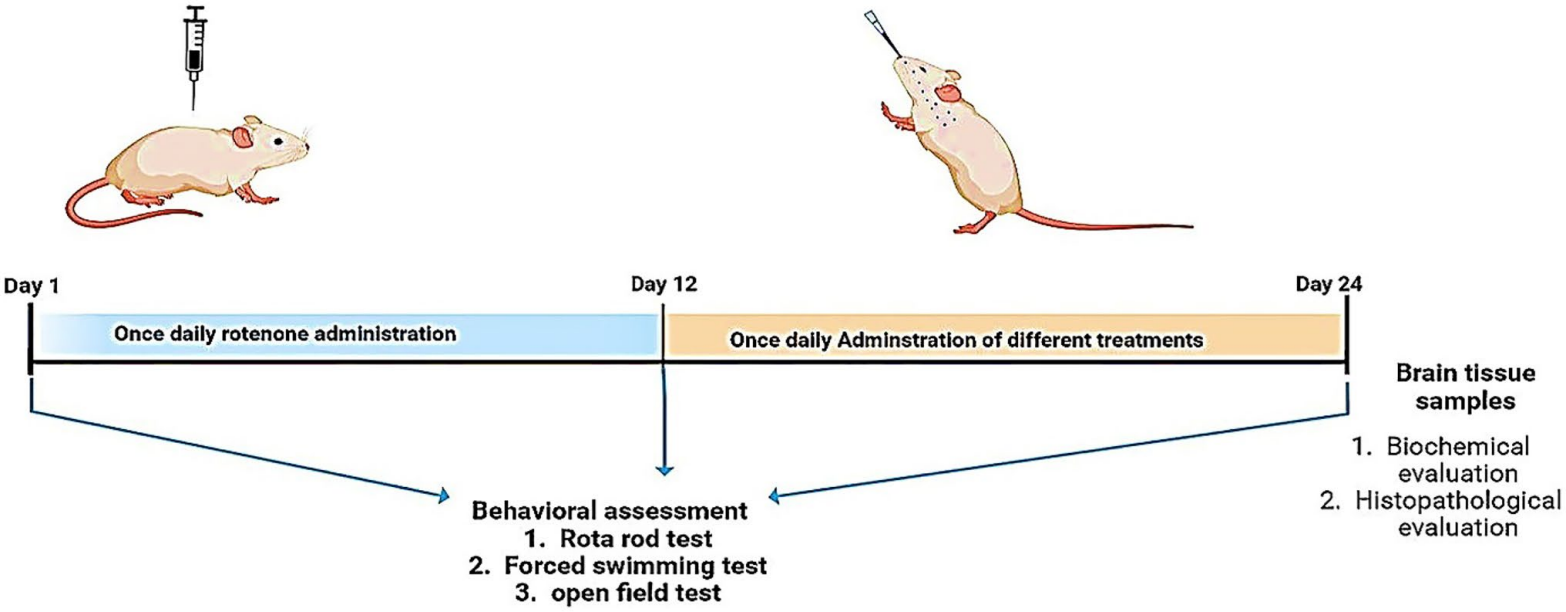
Experimental design and treatment schedule for the assessment of antiparkinsonian effects of the different prepared intranasal TAN-NLC formulations versus TAN suspension

Neurodegeneration simulating PD was induced in rats by subcutaneous injection (s.c.) of rotenone (2 mg/kg) daily for 12 consecutive days [1]. Rotenone solution (50 mg/ml) was freshly prepared by dissolving in dimethyl sulfoxide followed by further dilution to 2.5 mg/ml by mixing with 0.5% w/v aqueous solution of carboxymethyl cellulose sodium in a ratio of 1:20, respectively. The experimental design is summarized in Fig. 1. To confirm induction of the disease, various behavioral tests (rotarod test, forced swim test, and open field test) were carried out before and after rotenone administration. As well, these tests were repeated after treatment with various prepared formulations to assess their antiparkinsonian activity.

#### Treatment with different TAN-NLC formulations

Animals were randomly divided into five groups (*n* = *7*): negative control normal rats, positive control (untreated), TAN suspension in 0.5% w/v aqueous solution of carboxymethyl cellulose sodium, TAN-NLCs, and CS-TAN-NLCs. All the treatments were intranasally administered on daily basis for another subsequent 12 days following day 12 after rotenone s.c. injection. The technique involves installing the formulation (70 μl) to a depth of 13 mm in each nostril using a flexible cannula attached to a micropipette tip with keeping the animals in supine position for 30 s to prevent nasal leakage [25]. The once-daily dose was equivalent to 0.3 mg/kg TAN either in free form or loaded into different NLCs.

#### Behavioral tests

##### Rotarod test

Rota rod test was conducted to evaluate balance and motor coordination for tested animals. A 3-day training program was carried out for all rats to determine a steady baseline for their performance. After being trained, three different trials/day for each rat were performed using the rotarod’s accelerating speed mode (4 to 40 rpm) for 5 min. The latency to fall off the rotarod was recorded, and the results were expressed as mean of three trials for each rat [26].

##### Forced swim test

This test was done to assess the non-motor manifestations of PD such as depression. One day before the test, rats were trained by their placing in a water tank (20 × 20 × 40 cm) at 15 cm depth at 24 ℃ for 15 min. After that, they were subjected to the forced swim test for 5 min. The experiment was video-recorded and analyzed by the observer. Activity (swimming and climbing times) and immobility times were the parameters measured in this test and were considered an index for depression [26].

##### Open field test

This test was performed to investigate both locomotor and rear activities. An open-field wooden box (50 × 30 × 20 cm) that was divided into 25 squares was used. Each rat was placed on the box corner opposite to the wall followed by determination of the number of squares crossed in 3 min. The number of rears and latencies to rear and to move was recorded [26].

#### Brain tissue homogenization and biochemical assessment

At the end of the study, rats were sacrificed by decapitation, and then the striatum was dissected out and divided into two equal parts. One of them was stored at−80 °C till biochemical analysis. The other was used for histopathological and immunohistochemical evaluation.

Frozen striata from different animals were homogenized in PBS (1:10 w/v) and centrifuged at 2000 g for 15 min at 4 °C. The resultant supernatants were harvested for various biochemical assays.

##### Assessment of dopamine level

Striatal levels of dopamine (DA) were determined using rat dopamine ELISA Kit (Cat# CSB-E08660r, CUSABIO, USA) according to the manufacturer’s instructions and the results were expressed as ng/g tissue.

##### Assessment of inflammatory markers

The levels of nuclear factor kappa-β (NF-κβ) (Cat# MBS453975, MyBioSource, USA) and cathepsin B (Cat# LS-F9334, LSBIO, USA) in brain tissue homogenates were measured using ELISA method according to the manufacturer’s instructions.

##### Assessment of oxidative stress markers

Malondialdehyde (MDA) level was measured using a colorimetric method by thiobarbituric acid reaction [27]. Additionally, striatal glutathione (GSH) (Cat# E-BC-K030-M, Elabscience, USA) and hemoxygenase-1 (HO-1) (Cat# CSB-E08267r, CUSABIO, USA) levels were measured using an Eliza kit following the manufacturer’s instructions.

#### Histopathological examination

Brain tissue samples were fixed in a 10% formalin solution. Sections (5 μm thickness) were transversally cut and stained with hematoxylin and eosin (H&E) prior to microscopical examination using a light microscope (Olympus America Inc., USA)—equipped with spot digital camera (16-bit digital camera (1280 × 1024)).

#### Immunohistochemical analysis of tyrosine hydroxylase (TH)

After fixation of brain tissue samples in 10% formalin solution, sections of 4 µm thickness were cut, dried overnight at 37 °C, and then deparaffinized and rehydrated. Sections were stained using tyrosine hydroxylase (TH) polyclonal antibodies (Cat# PA5-85,167, Invitrogen, Thermo-Fischer Scientific, USA). After incubation with both primary and secondary antibodies, an ultra-vision one HRP polymer and diaminobenzidine (DAB) kit components were used to complete the staining process (Cat# PA5-85,167, Invitrogen, Thermo-Fischer Scientific, USA). Hematoxylin was used as a counterstain followed by microscopical examination of the stained sections [28].

### Statistical analysis

All experiments were performed in triplicates and results were expressed as mean ± SD. Statistical analysis was carried out by one-way analysis of variance (ANOVA) followed by a post hoc Tukey’s test for multiple comparisons using GraphPad Prism (Version 7.04, San Diego, CA, USA). The level of significance was set at *p* ≤ *0.05*.

## Results and discussion

### Screening of various oils and solid lipids

The poor bioavailability of the lipophilic TAN prompts the development of a lipid-based nanocarrier. For better drug loading, NLCs were preferred over solid lipid nanoparticles. Screening of various solid and liquid lipids is considered an important pre-formulation step to select the best ingredients achieving the maximum drug solubility. Results revealed that TAN solubility in Compritol, Glyceryl monostearate (GMS), and Precirol were 20.3 ± 0.62, 16.2 ± 1, and 12.9 ± 0.4 mg/g, respectively. Therefore, Compritol was selected in the current study as the solid lipid for preparation of NLCs. As for the liquid lipids, TAN solubility was in the following order: Peceol (3.2 ± 0.2 mg/g) > Labrafac lipophile = Labrafac PG (1.8 ± 0.2 mg/g). Although Peceol achieved higher drug solubility than Labrafac lipophile, it was not selected for preparation of NLCs because Peceol leads to formation of unstable nanosystem with Compritol as detected by phase separation of the formed emulsion.

### Preparation of TAN-NLCs

In the present study, TAN-NLCs were prepared by using melt-emulsification ultra-sonication method which is preferred over other methods utilizing the use of toxic organic solvents (solvent-emulsification evaporation and solvent-emulsification diffusion) [29]. This technique allowed for the production of smaller NLCs with low polydispersity and high drug loading. Additionally, it was reproducible and easy for scaling up. Compritol and Labrafac lipophile were chosen as solid and liquid lipids in a ratio of 3:2 w/w, respectively. Based on the results, it was suggested that the used surfactant mixture (Kolliphor^®^ HS15 and Tween^®^ 80, 3:2 w/w, respectively) was efficient to maintain the colloidal stability of the prepared NLCs and prevent their aggregation along with increasing drug loading. Furthermore, the use of Tween^®^ 80 was reported to increase brain accumulation after non-invasive intranasal administration [30]. TAN loading was simply achieved by dissolving the drug in the lipid phase to achieve a concentration of 0.5 mg/ml of final formulation volume.

### Preparation and optimization of CS-TAN-NLCs

CS has been extensively studied as a natural cationic mucoadhesive polymer enhancing nose-to-brain drug delivery via increasing drug nasal residence time. Additionally, it was reported to have antioxidant and neuroprotective effect [19, 20]. Therefore, modification of the surface of the developed TAN-NLCs with CS was performed hoping to improve the antiparkinsonian potential of TAN. To minimize the need for tedious and time-consuming washing steps to remove excess chitosan, a simple titration technique was adopted to prepare CS-TAN-NLCs. It is necessary to optimize the amount of chitosan added to achieve uniform coatings, as excess chitosan may affect NLCs size and homogeneity [22]. Consequently, three different concentrations of chitosan solution (0.1%, 0.2%, 0.5% w/w) in 1% v/v acetic acid were tried with three different ratios: 1:10, 2:10, and 3:10 v/v chitosan solution to TAN-NLCs, respectively. Additionally, mild heating at 37 °C for 1 h was conducted for efficient coating. This could be attributed to the ability of elevated temperature to change the brush confirmation of the bulky PEG chains of Kolliphor^®^ HS15 (present on the surface of NLCs), resulting in better penetration of the inserted polymeric coat between these chains [12].

The effect of adding different volumes of different chitosan concentrations on particle size, PDI, and ζ-potential of the coated TAN-NLCs would be demonstrated later in Table 1 (the “Colloidal properties for optimization of different CS-TAN-NLCs” section).

**Table 1:** Effect of addition of different chitosan (CS) concentrations and different CS: TAN-NLCs ratios on the colloidal properties of different prepared CS-TAN-NLCs

**Formulation code**	**CS concentration (% w/v)**	**CS: TAN-NLCs** (**v/v)**	**Particle size (nm)**	**PDI**	**ζ-potential (mv)**
**F1**	0	-	152.2 ± 2.9	0.2 ± 0.01	−14.4 ± 0.2
**F2**	0.1	1:10	178.0 ± 2.8	0.8 ± 0.01	−1.7 ± 0.3
**F3**	0.1	2:10	190.1 ± 2.4	0.78 ± 0.04	2.2 ± 0.9
**F4**	0.1	3:10	245.0 ± 6.2	0.47 ± 0.03	7.9 ± 0.3
**F5**	0.2	1:10	183.4 ± 0.9	0.46 ± 0.04	8.3 ± 0.4
**F6**	0.2	2:10	194.4 ± 3.0	0.41 ± 0.02	9.8 ± 0.5
**F7**	0.2	3:10	236.7 ± 6.9	0.34 ± 0.01	17.3 ± 0.6
**F8**	0.5	1:10	196.1 ± 1.0	0.32 ± 0.01	20.8 ± 0.8
**F9**	0.5	2:10	245.3 ± 3.5	0.34 ± 0.02	26.0 ± 0.7
**F10**	0.5	3:10	291.3 ± 1.5	0.33 ± 0.01	29.4 ± 0.5

Data are expressed as mean ± SD (*n* = *3*); average EE% of all prepared formulations was ~98 ± 1.2%

### Physicochemical characterization

#### Colloidal properties for optimization of different CS-TAN-NLCs

As shown in Table 1, The uncoated TAN-NLCs exhibited good colloidal properties: size 152.2 ± 2.9 nm, PDI 0.2 ± 0.01, and ζ-potential−14.4 ± 0.2 mV. The slight negative ζ-potential of TAN-NLCs could be ascribed to the presence of some acidic groups in the solid lipid Compritol used in their preparation [31]. Additionally, the existence of traces of unesterified fatty acids in Tween^®^ 80 [32] together with the formation of intramolecular hydrogen bonding in Kolliphor^®^ HS15 produces a negative surface charge [8]. Addition of different ratios of CS:TAN-NLCs at each tested CS concentration resulted in a gradual increase in size along with charge inversion from the negative to the positive side ensuring efficient coating process (Table 1). Our results matched those previously mentioned for various CS-coated NLCs [33, 34] or solid lipid nanoparticles [35]. The cationic nature of CS is conferred by the abundant acetylated and deacetylated amino groups present in its structure [36]. Coating TAN-NLCs with CS occurs through electrostatic interaction between CS and the negatively charged TAN-NLCs. Additionally, it was observed from Table 1 that the homogeneity of size distribution for the different prepared formulations increased with increasing chitosan concentration as indicated by the measured PDI reaching ~0.3 for CS concentration 0.5% w/v. This could be assigned to possessing the highest positive ζ-potential values (20.8–29.4 mV) and hence lower tendency for aggregation with higher colloidal stability. Among different formulations tested, F8 with 0.5% w/v CS concentration and ratio 1:10 CS to TAN-NLCs was selected as an optimum formulation for further in vitro and in vivo characterization. The selection was based on having favorable colloidal properties (size < 200 nm, PDI ~0.3, and ζ-potential 20.8) along with using lower ratio CS:TAN-NLCs to avoid excessive dilution of the drug in the loaded formulation.

#### TAN entrapment efficiency (EE)

Both TAN-NLCs and CS-TAN-NLCs formulations displayed high drug EE of 98.1 ± 0.7% and 98.7 ± 0.6%, respectively. This could be due to the lipophilic nature of TAN and its solubility in the lipid matrix of NLCs. Furthermore, NLCs were reported to efficiently trap lipophilic drugs as they contain liquid lipids with the solid lipids that cause structural imperfections in the solid lipids. Accordingly, disturbance in crystalline arrangement of solid lipids takes place and hence promoting high drug loading and preventing drug leakage [37]. Moreover, it was found that chitosan coating of NLCs did not significantly (*p* > 0.05) affect drug EE, an observation previously stated for quercetin loaded in CS-coated NLCs [38].

#### Microscopical examination

**Fig. 2:**
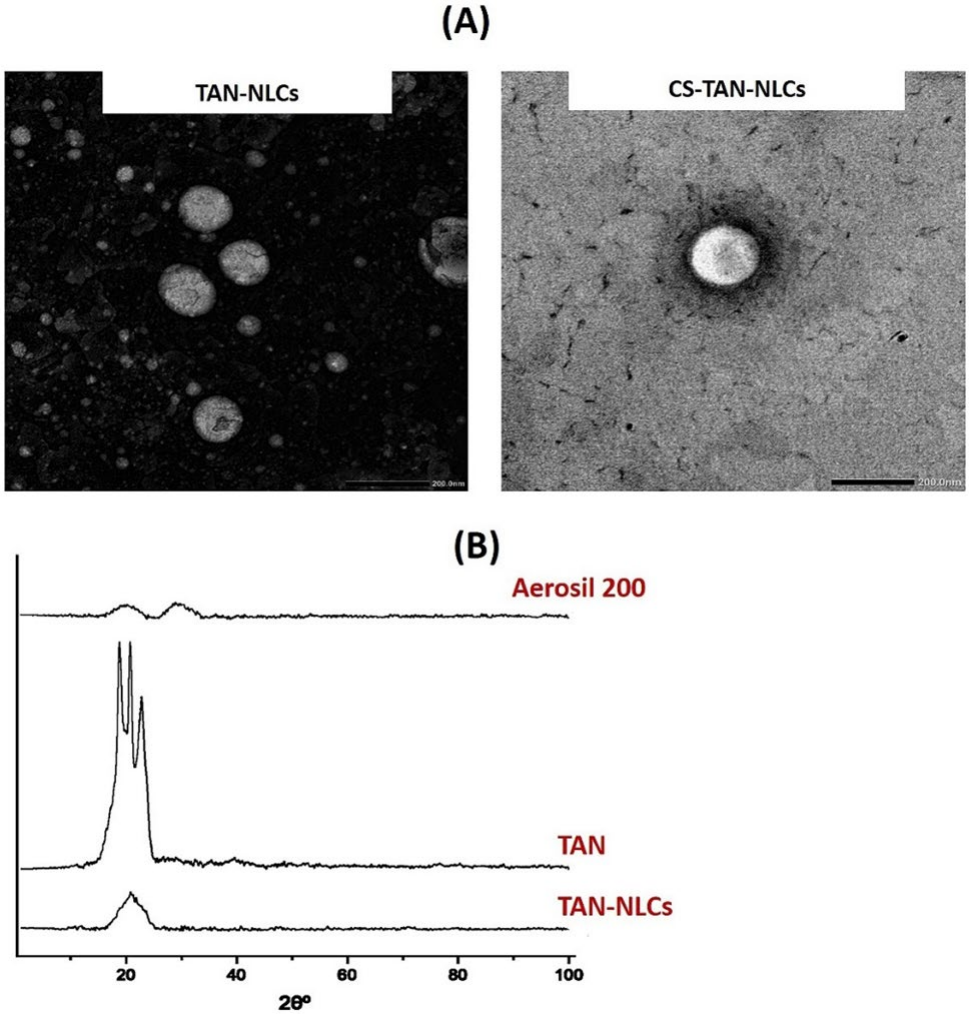
**A** TEM images of different TAN-NLC formulations. Magnification × 20K, scale bar represents 200 nm. **B** X-ray diffraction pattern of TAN and TAN-NLCs

TEM imaging of both uncoated and coated TAN-NLC formulations presented the typical appearance of NLCs with morphological features similar to those described earlier [29, 36] (Fig. 2A). The prepared formulations appeared spherical in shape with size < 200 nm and homogenous size distribution, further confirming the results obtained by the Zetasizer. Additionally, no TAN crystals were found during TEM examination, further confirming efficient drug entrapment in the prepared NLC formulations.

#### X-ray diffractometry (XRD)

To investigate TAN crystallinity in the developed TAN-NLCs, nondestructive XRD was carried out. Figure 2B showed the XRD patterns of TAN and TAN-NLCs along with Aerosil^®^ 200 that was used for drying aqueous dispersions of tested NLCs. Aerosil^®^ 200 diffractogram confirmed its amorphous structure, since a broad peak at 22.5° was only detected [29]. TAN diffractogram presented prominent sharp diffraction peaks between 5 and 30°, emphasizing its crystallinity [8]. The characteristic drug peaks disappeared in TAN-NLCs diffractogram indicating its amorphous state as the drug was molecularly dispersed in the lipid matrix of NLCs. The obtained findings were in agreement with those previously reported where entrapment of albendazole [39] or fenofibrate [40] in the prepared CS-coated NLCs resulted in disappearance of all drug characteristic diffraction peaks in the prepared NLCs diffractogram.

#### In vitro drug release

**Fig. 3:**
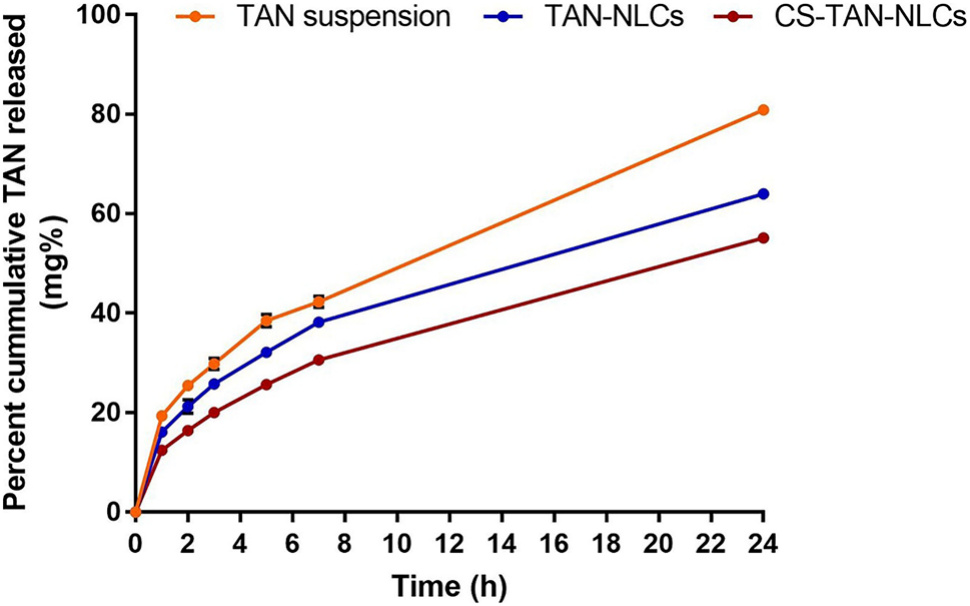
Release profile of TAN from TAN-NLCs and CS-TAN-NLCs formulations versus TAN-suspension in 50% ethanolic water at 37 °C for 24 h (*n* = 3)

In vitro drug release profile of TAN suspension and different NLCs formulations (TAN-NLCs and CS-TAN-NLCs) is shown in Fig. 3. TAN suspension showed a significantly higher burst release ~20% after 1 h compared to both TAN-NLCs (~16%) and CS-TAN-NLCs (~12%) (*p* ≤ *0.05*). Additionally, the coated formulation showed a significantly lower burst release at 1 h compared to the uncoated one (*p* ≤ *0.05*), indicating the role of the chitosan coat in slowing down TAN release rate. The burst release of TAN from different formulations could be ascribed to the unentrapped drug adsorbed on the surface of prepared NLCs rather than being incorporated in lipid matrix [29]. Whereas after 24 h, Free TAN showed almost complete and significantly higher release with ~80% compared to only ~64% and ~55% from TAN-NLCs and CS-TAN-NLCs, respectively (*p* ≤ 0.001). This observed delayed release profile of TAN was in line with previous studies in which a sustained drug release from NLCs was credited to the slow diffusion of the drug that was efficiently solubilized and encapsulated in the lipid matrix [41]. Furthermore, the lower % TAN released for CS-TAN-NLCs as compared to TAN-NLCs could be due to the additional barrier formed by the coating layer limiting the diffusion of the release medium into the NLCs matrix [38]. It is worth noting that the delayed release of the developed CS-TAN-NLCs over 24 h was beneficial to increase the residence time of the drug in the brain allowing for once daily administration since it was reported that the intranasally administered drugs were completely cleared from CNS after ~4 h [42].

The drug release mechanism of TAN-NLCs and CS-TAN-NLCs was determined by fitting to different release kinetics models: zero-order, first-order, Higuchi, Korsmeyer-Peppas, and Hixson Crowell models. The results designated diffusion-controlled TAN release from both TAN-NLCs and CS-TAN-NLCs as the largest coefficient of determination (R^2^) value (0.999) and the smallest mean standard error (MSE) value (~0.1) were detected for the Korsmeyer-Peppas model. A release exponent value (*n*) ≤ 0.5 was noticed reflecting Fickian diffusion.

#### Shelf stability study

Table 2 demonstrated the effect of storage on the colloidal properties in addition to TAN % EE for TAN-NLCs and CS-TAN-NLCs at 4 °C for 3 months. Visual examination of the tested formulations reflected the stability of the developed formulations as no drug precipitation was observed upon storage. The insignificant differences in the evaluated physicochemical parameters substantiated the stability of the tested formulations for 3 months at 4 °C. Similar results were reported previously for long-term shelf stability of the nose-to-brain delivered chitosan-coated NLCs encapsulating buspirone up to 6 months at both 5 and 25 °C [43]. Additionally, Pokharkar et al. [44] proved the 3-month stability of their prepared rosuvastatin-loaded NLCs against aggregation and drug leakage. They monitored the prepared NLCs by microscopical examination monthly throughout the study period and revealed no signs of drug leakage or change in the size [44].

**Table 2:** Storage stability data of TAN-NLCs and CS-TAN-NLCs at 4 °C for 3 months (*n* = *3*)

**Time (month)**	**Formulation**	**Particle size (nm)**	**PDI**	**ζ-potential (mv)**	**%EE**
**0**	TAN-NLCs	152.2 ± 2.9	0.2 ± 0.01	−14.4 ± 0.2	98.1 ± 0.7
	CS-TAN-NLCs	196.1 ± 1.0	0.32 ± 0.01	20.8 ± 0.8	98.7 ± 0.6
**1**	TAN-NLCs	149.3 ± 3.1	0.22 ± 0.009	−13.6 ± 1	98.4 ± 0.9
	CS-TAN-NLCs	200.2 ± 5.6	0.34 ± 0.011	21.3 ± 1.1	98.3 ± 0.04
**2**	TAN-NLCs	150.6 ± 2.8	0.21 ± 0.015	−15.3 ± 0.9	98.2 ± 0.04
	CS-TAN-NLCs	198.7 ± 4.1	0.31 ± 0.013	22.4 ± 1.2	98.6 ± 0.03
**3**	TAN-NLCs	151.5 ± 1.4	0.23 ± 0.02	−14.1 ± 0.6	98.8 ± 0.06
	CS-TAN-NLCs	199.2 ± 4.5	0.33 ± 0.015	21.9 ± 1.8	99.0 ± 0.03

### In vivo assessment of the antiparkinsonian activity

Rotenone had been extensively studied in numerous preclinical research to cause neurodegeneration and hence PD-like symptoms in rodents [1, 45, 46]. Subcutaneous rotenone administration was reported to cause damage to the dopaminergic neurons in substantia nigra of rodents through generation of reactive oxygen species (ROS) along with inhibition of mitochondrial respiration [47, 48]. Accordingly, a rat model of PD was conducted in the present study via s.c injection of rotenone and the antiparkinsonian activity of the different developed intranasal TAN-loaded uncoated and chitosan-coated NLCs vs TAN suspension was assessed through many behavioral tests and biochemical and histopathological evaluation.

#### Behavioral tests

Table 3 demonstrated the results of the rotarod test that was performed for assessment of rats’ motor function. Results revealed that positive control rats displayed a significantly decreased latency to fall time compared to negative control (17.8 ± 2.3 s vs 141.7 ± 20.1 s, *p* ≤ 0.001, respectively), indicating the ability of the injected rotenone to induce motor deficit, that denoted parkinsonism behavior. In contrast, intranasal TAN either in free form or loaded into different NLCs formulations brought about significant prolongation in time to fall vs the positive control (*p* ≤ 0.001) in the following order: TAN suspension < TAN-NLCs < CS-TAN-NLCs. It is noteworthy that only CS-TAN-NLCs succeeded to restore the normal locomotive function as presented by its statistical similarity with the negative control (*p* > 0.05), demonstrating its ability in preventing loss of dopaminergic neurons [19, 49].

**Table 3:** Effect of tested TAN-NLC formulations and TAN suspension on different behavioral and locomotor tests after intranasal administration (0.3 mg/kg) to rotenone-induced PD rat model

**Parameter**	**Negative control**	**Positive control**	**TAN suspension**	**TAN-NLCs**	**CS-TAN-NLCs**
**Rotarod test**					
Mean latency to fall (s)	141.7^**a**^ ± 20.1	17.8^**d**^ ± 2.3	70.1^**c**^ ± 2.8	92.1^**b**^ ± 5.8	129.2^**a**^ ± 21.6
**Open field**					
Latency to move (s)	4.5^**d**^ ± 0.5	10.1^**a**^ ± 0.5	7.4^**b**^ ± 0.3	6^**c**^ ± 0.3	4^**d**^ ± 0.4
Number of crossed squares	70.2^**a**^ ± 3.9	24.2^**d**^ ± 5	51.5^**c**^ ± 5.4	60.1^**b**^ ± 2.5	67.3^**a**^ ± 3
Latency to rear (s)	10.5^**c**^ ± 1.9	16.4^**a**^ ± 1	14.3^**b**^ ± 0.9	11.3^**c**^ ± 0.4	9.8^**c**^ ± 1.5
Number of rears	18.5^**a**^ ± 3	7^**c**^ ± 1.4	12.5^**b**^ ± 1	16^**a**^ ± 1.4	18.3^**a**^ ± 2.7
**Forced swimming test**					
Activity time (s)	162.5^**a**^ ± 4.2	98.8^**e**^ ± 3.3	140^**d**^ ± 2.1	132.5^**c**^ ± 2.4	157^**b**^ ± 3.8
Immobility time (s)	17.5^**e**^ ± 4.23	81.2^**a**^ ± 3.3	47.5^**b**^ ± 2.4	40^**c**^ ± 2.1	23^**d**^ ± 3.9

The study was conducted on male Wistar rats (*n* = 7). Values were expressed as means ± SD. Data was analyzed using one-way ANOVA followed by post hoc test (Tukey) for group comparisons. Means of similar symbols are statistically insignificant: a > b > c > d > e (*p* ≤ 0.001)

Regarding open field test, the motor activity of rats exposed to rotenone exhibited a significant delay to either move or rear besides a significant decrease in the number of crossed squares and rears compared to the negative control (*p* ≤ 0.001) (Table 3). Conversely, different treatments gave rise to a significant increase in the mobility time along with the number of crossed squares and rears compared to the positive control group (*p* ≤ 0.001). Again, the greatest efficacy was achieved by the coated formulation as indicated by absence of statistical difference with the negative control in the determined parameters (*p* > 0.05) elucidating its role in reversal of induced motor disability.

As for the forced swim test, rotenone-treated rats displayed a significant delay in activity time together with a prolongation in immobilization compared with negative control and different treated animals (*p* ≤ 0. 001) (Table 3). These results designated the depressive-like behavior of rotenone, which was improved by different treatments and coated formulation displayed the most prominent effect.

Behavioral testing results inferred that free TAN was effective in improving motor and depressive manifestations of PD which was consonant with previous reports, and was mainly due to its neuroprotective potential being a potent antioxidant [49, 50]. The antiparkinsonian activity of TAN was significantly augmented after its loading into NLCs, suggesting effective brain delivery. This could be attributed to the presence of the drug in a solubilized form in the nanocarrier matrix. Therefore, achieving successful brain delivery as free drug suspension was reported to be retained in the olfactory bulb with limited nose-to-brain drug delivery [51]. Additionally, free drug might be exposed to either nasal metabolizing enzymes or nasal mucociliary clearance, thereby subjected to rapid elimination before reaching its target site (brain) [52]. Another advantage of NLCs is that being of nanosize, they can easily be transported either transcellular by olfactory neurons or by different endocytic pathways of neuronal cells leading to an increase in drug brain bioavailability [53].

It is noteworthy that the elaborated CS-TAN-NLCs had the greatest antiparkinsonian activity with restoration of motor function back to normal owing to its mucoadhesive properties and hence increasing nasal retention, together with the therapeutic effect of CS as neuroprotective and antioxidant agent [19, 20]. Mucoadhesive characteristics of the developed CS-TAN-NLCs were mainly attributed to its positive surface charge which allowed for effective electrostatic attraction to the anionic sialic groups present on the nasal mucosa [54].

#### Assessment of striatal dopamine level

**Fig. 4:**
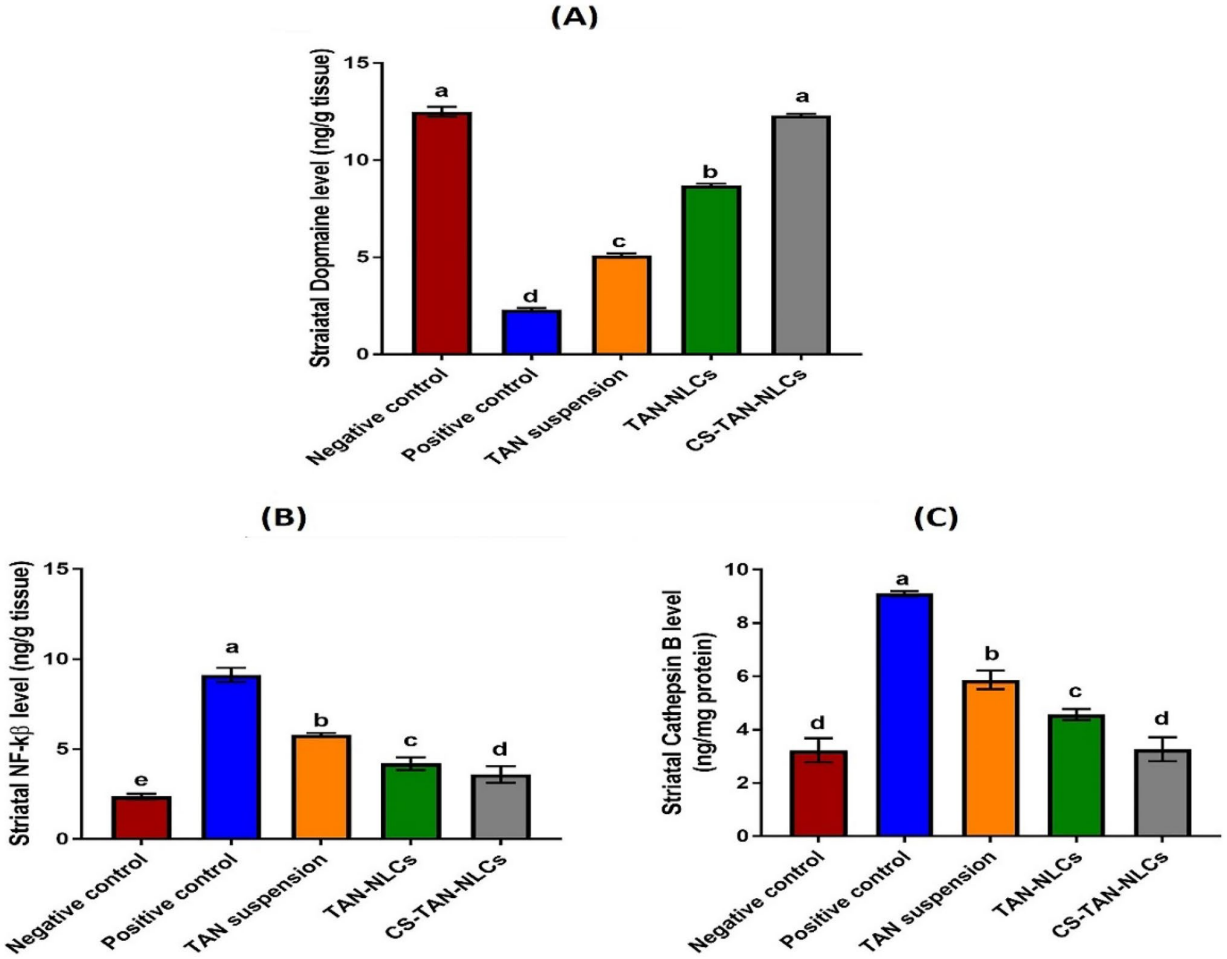
Evaluation of striatal **A** dopamine as well as inflammatory markers, **B** NF-kβ, and **C** cathepsin B in different treatment groups. Data are expressed as mean ± SD (*n* = 7) and analyzed using one-way ANOVA followed by post hoc test (Tukey) for group comparisons. Means of similar symbols are statistically insignificant: a > b > c > d > e (*p* ≤ 0.001)

Loss of dopaminergic neurons in the substantia nigra plays a key role in the pathophysiology of PD [1]. Therefore, the dopamine level in brain homogenates of different tested groups was determined using an Eliza assay method and the result is presented in Fig. 4A. It was found that the dopamine level was significantly reduced in the positive control group (2.3 ± 0.1 ng/g tissue) in comparison to the negative control one (12.5 ± 0.25 ng/g tissue) (*p* ≤ 0.001). Treatment with TAN in the form of intranasal suspension and TAN-NLCs moderately restored brain dopamine levels to 5.1 ± 0.11 and 8.7 ± 0.09 ng/g tissue, respectively. It was reported that administration of TAN in rats with PD prevented damage of nigrostriatal dopaminergic neurons and hence elevated the level of striatal dopamine [6]. Restoration of normal DA levels was observed for the CS-TAN-NLCs-treated group (12.3 ± 0.1 ng/g tissue) with absence of significant difference with negative control while achieving a 5.3-fold significant increase when compared to positive control, reflecting higher protection of dopaminergic neurons (*p* ≤ 0.001).

#### Assessment of the inflammatory markers

As reported previously, neuroinflammation and oxidative stress are considered main contributors to PD pathogenesis [55]. Rotenone systemic administration can cause activation of the brain’s resident immune cells (microglia). Activated microglial cells produce numerous inflammatory mediators via activation of NF-kβ signaling pathway. NF-kβ is a transcriptional factor that plays a crucial role in induction of inflammation via regulation of genes encoding inflammatory cytokines [56]. NF-kβ-mediated neuroinflammation plays a key role in the pathogenesis of PD via promoting dopaminergic neurons loss and increase in α-synuclein fibrillation [57]. Furthermore, microglial activation leads to production of cathepsin B, a lysosomal cysteine protease. It was reported that increased activation of NF-kβ may increase the expression of microglial cathepsin B. Additionally, increased cathepsin B further activates NF-kβ through the autophagic system in microglia. Consequently, increased levels of cathepsin B can lead to production of proinflammatory mediators, generation of mitochondria-derived ROS, and eventually induction of neuronal death [58, 59].

In this context, the striatal levels of both NF-kβ and cathepsin B were quantitively determined to investigate the possible molecular pathway for the antiparkinsonian activity of TAN. As shown in Fig. 4B, C, administration of rotenone in positive control group resulted in induction of severe neuroinflammation as verified by the significant elevation in the striatal levels of both NF-kβ and cathepsin B by 3.8-fold and 2.8-fold, respectively, compared to the negative control group (*p* ≤ 0.001). Different treatments significantly decreased the tested markers compared to the positive control group (*p* ≤ 0.001). TAN suspension group revealed 36.5% and 35.5% decrease in the levels of both NF-kβ and cathepsin B, respectively, versus the positive control group (*p* ≤ 0.001) (Fig. 4B, C). This was ascribed to the pronounced anti-inflammatory activity of TAN which has been previously described in different preclinical PD models. TAN can inhibit neuroinflammation and hence provide a neuroprotective effect [60]. Although the inhibitory effect of TAN on NF-kβ activation in activated microglia was previously reported in literature [50], investigation of its anti-inflammatory effect on cathepsin B expression was performed for the first time in the present study.

Regarding TAN-NLCs group, levels of both NF-kβ and cathepsin B were significantly decreased by ~54% and ~50%, respectively, compared with positive control group (*p* ≤ 0.001), confirming the role of NLCs in increasing TAN brain bioavailability (Fig. 4B, C). Interestingly, CS-TAN-NLCs significantly achieved a higher reduction in NF-kβ and Cathepsin B striatal levels by 14.3 and 28.4% versus uncoated TAN-NLCs (*p* ≤ 0.01), reflecting superior efficacy (Fig. 4B, C).

#### Assessment of oxidative stress markers

It is extensively reported that oxidative stress is one of the crucial factors contributing to PD [55]. It is produced by an imbalance between the generation and elimination of ROS and consequently formation of malondialdehyde (MDA).

Glutathione (GSH) is an antioxidant tripeptide which plays a crucial role in the manifestation of neurodegenerative diseases such as PD [1].

Heme oxygenase-1 (HO-1) was reported to be involved in the pathophysiology of PD [61]. It is a cellular protein that catalyzes the conversion of heme into biliverdin, carbon monoxide, and free ferrous iron in brain and other tissues. Despite having a neuroprotective role via the antioxidant properties of biliverdin and bilirubin in some studies, others reported that chronic overexposure of HO-1 may induce intracellular oxidative damage in mitochondria and other subcellular organelles via heme-derived iron and carbon monoxide. Therefore, its level is elevated in PD being a chronic neurodegenerative condition [62]. On this basis, upregulation of HO-1 was also accompanied by loss of dopaminergic neurons in a rat model of PD [62].

**Fig. 5:**
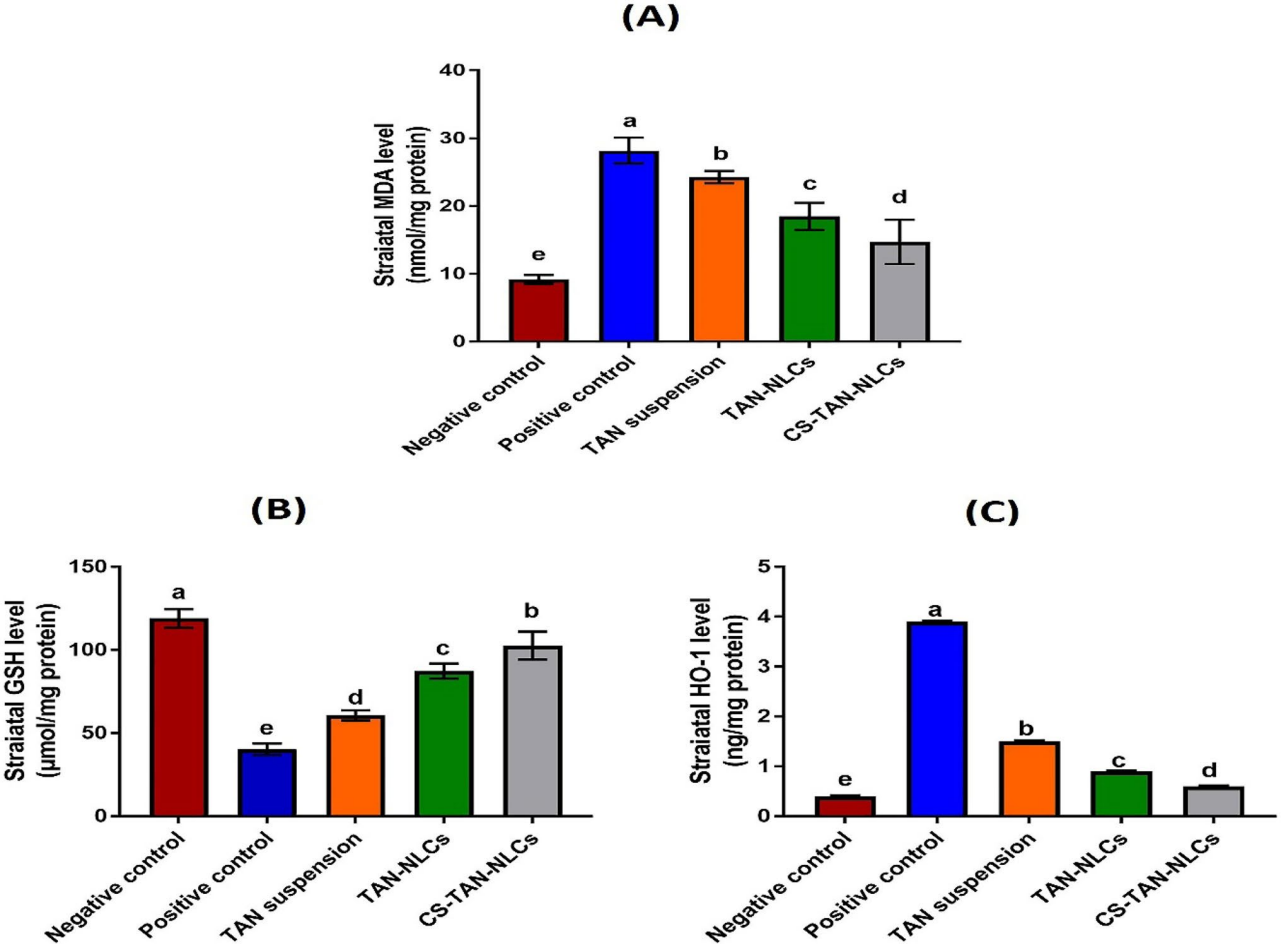
Effect of intranasal administration of different treatments (0.3 mg/kg) on the measured oxidative stress markers; **A** malondialdehyde, **B** glutathione, and **C** heme oxygenase-1. Values are presented as mean ± SD (*n* = 7). Data are analyzed using one-way ANOVA followed by post hoc test (Tukey) for group comparisons. Means of similar symbols were statistically insignificant: a > b > c > d > e (*p* ≤ 0.001)

Therefore, the aforementioned oxidative stress markers (MDA, GSH, and HO-1) were determined in the current work and the results were presented in Fig. 5A–C. Rotenone administration in the positive control group demonstrated a significant increase in striatal MDA and HO-1 levels by ∼ 3.1-fold and ∼9.8-fold, respectively, together with a significant decrease in the levels of the antioxidant GSH by ∼66% compared to the negative control (*p* ≤ 0.001). This is assigned to the well-documented ability of rotenone to induce oxidative stress in brain mimicking the natural age-related decrease in the brain antioxidant defense system [1].

Intranasal administration of various formulations caused a significant decline in levels of both MDA and HO-1 besides a significant restoration of GSH levels, compared to the positive control (*p* ≤ 0.001). The detected improvement in the levels of the tested markers for TAN suspension group (~50% increase in GSH with ~14% and 61.5% decrease in MDA and HO-1, respectively, versus positive control) was mainly owing to its reported potent antioxidant potential [50]. Treatment with TAN-NLCs succeeded to decrease MDA and HO-1 by ~34% and 77% as well as increase in GSH by 116.5% versus positive control. The observed mitigation of the induced oxidative stress was attributed to the ability of the NLCs to increase TAN brain bioavailability via its solubilization in a fine molecular state together with protection against nasal metabolism. Although TAN suspension and TAN-NLCs achieved a significant improvement compared to the positive control group (*p* ≤ 0.001), they were still significantly inferior to CS-TAN-NLCs. The latter exhibited a significant elevation by 154.6% in GSH levels with ∼48% and 84.6% significant reduction in the levels of both MDA and HO-1 compared to the positive control (*p* ≤ 0.001).

#### Histopathological examination

**Fig. 6:**
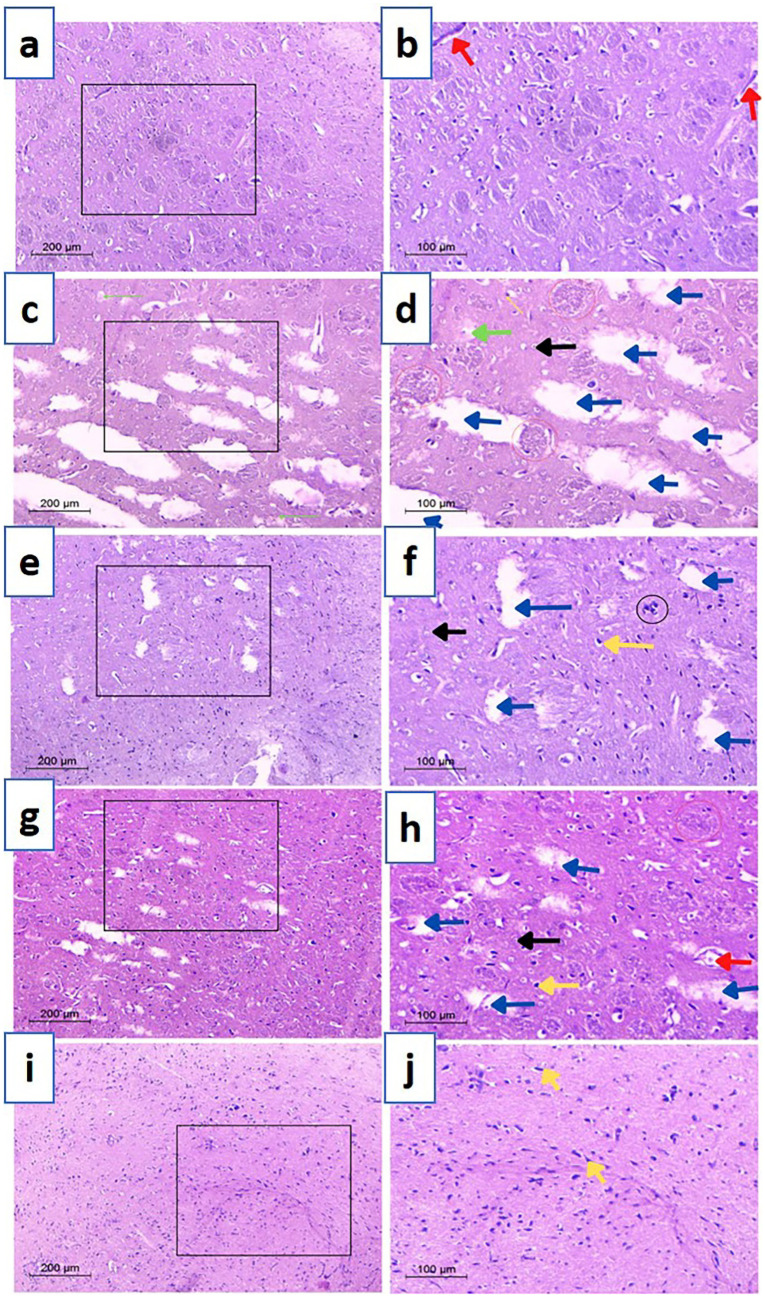
Microscopical images of rat striatal tissues stained with H&E. **a** and **b** Negative control, **c** and **d** positive control, **e** and **f** TAN-suspension, **g** and **h** TAN-NLCs, and **i** and **j** CS-TAN-NLCs. Black arrow (normal neurons); red arrow (blood vessel); blue arrows (cerebral infracts); red circles (eosinophilic plaques); green arrows (Lewy bodies); yellow arrows (pyknotic nuclei); black circle (DNA fragments). The right panel signifies a higher magnification of the area selected in the left panel. Magnification × 100, scale bar 200 µm (low)- × 200, scale bar 100 µm (high)

**Fig. 7:**
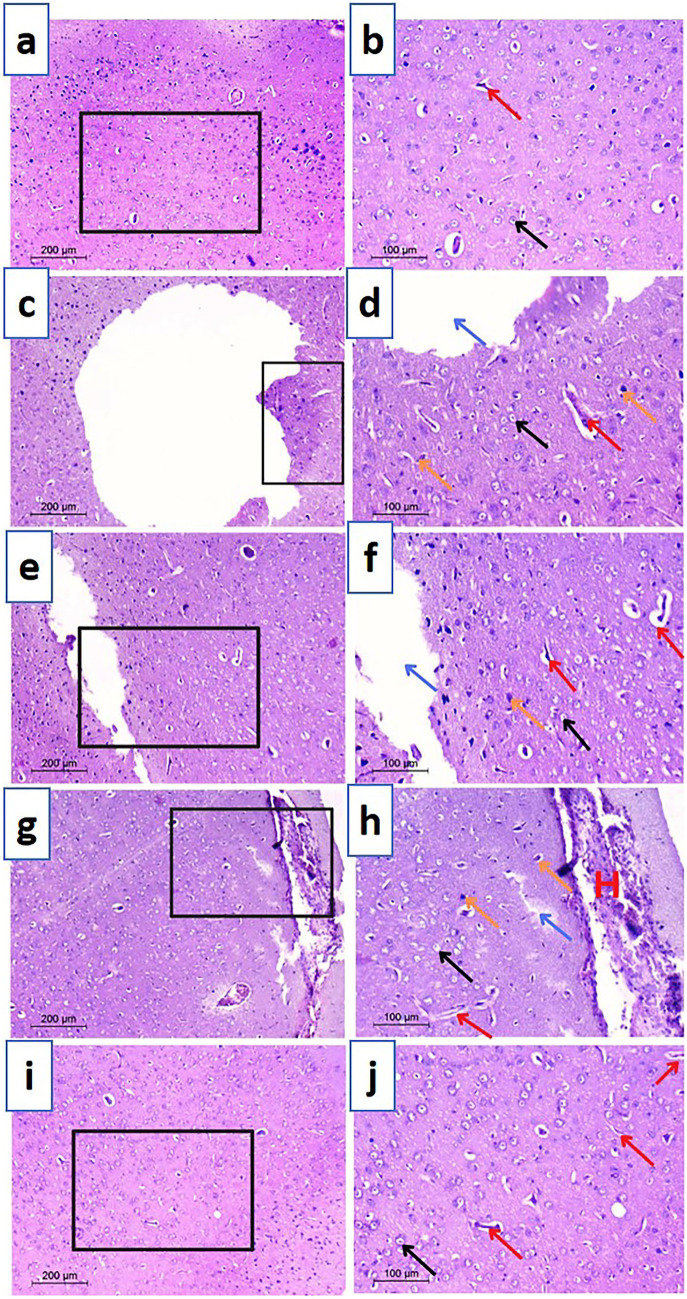
Microscopical images of examined prefrontal cortex sections from different treated rats stained with H&E. **a** and **b** negative control, **c** and **d** positive control, **e** and **f** TAN-suspension, **g** and **h** TAN-NLCs, and **i** and **j** CS-TAN-NLCs. Black arrow (normal neurons); red arrow (blood vessel); blue arrows (cerebral infracts); orange arrows (pyknotic nuclei); H (hemorrhage). The black box in the left panel highlighted the magnified part in the right panel. Left panel magnification is × 100, scale bar represents 200 μm while right panel magnification is × 200, the scale bar represents 100 μm

Findings of behavioral and biochemical evaluation were additionally verified via histopathological examination of excised striata and prefrontal cortices (Figs. 6 and 7). Examined striatal sections from negative control groups (Fig. 6a, b) demonstrated normal construction with characteristic architecture and cell size properties of healthy tissue. Besides having plenty of cytoplasm, neurons had spherical basophilic nuclei with some Nissl granules located peripherally. Furthermore, normal vascularization of the striatal was observed tissue indicating healthy structure was also present.

In contrast, induction of PD through rotenone administration resulted in significant neurological changes as shown in Fig. 6c, d. Examined sections revealed several multiple aberrant neurons evidenced by the presence of nuclear pyknosis and degeneration associated with gliosis and neuropil vacuolation. Focal numerous eosinophilic plaques and Lewy bodies were also developed. Additionally, neural tissue appears perforated due to severe tissue degradation (large infarcts).

Compared to the positive control group, TAN suspension group showed moderate histopathological improvements and reduced neurodegenerative symptoms (Fig. 6e, f). However, striatal infarcts, nuclear pyknosis, and necrotic symptoms were found with a greater prevalence of deteriorating neurons compared to other treatment groups, suggesting inferior efficacy. On the other hand, treatment with different TAN-loaded NLC formulations displayed variable effectiveness. In this regard, TAN-NLCs achieved a lower efficacy, as designated by the existence of few degenerative infarcts, but some degenerative changes characterized by presence of pyknotic nuclei with dilation of blood vessels and a low number of localized eosinophilic plaques were still seen (Fig. 6g, h). Interestingly, CS-TAN-NLCs showed the greatest antiparkinsonian potential as indicated by the normal appearance of the striatal neurons. Glial cells were flattened with normal neuropil along with complete absence of plaque tangles (Fig. 6i, j). The later finding further highlighted the importance of chitosan coating in augmenting the efficacy of TAN-NLCs.

Histopathological examination of prefrontal cortex sections was additionally performed to investigate the effect of induced neurodegeneration on the development of depressive symptoms in rats. Examined sections from different groups provided a similar analogy to the striatum. In this context, sections of the control group revealed normal neurons, with defined nuclei, receiving blood from multiple blood vessels (Fig. 7a, b). Conversely, a massive cerebral infarction and many dilated blood vessels were encountered in the rotenone treated group, reflecting a marked neuronal damage (Fig. 7c, d). The presence of pyknotic neurons also showed rotenone neurotoxicity. TAN suspension group demonstrated presence of neuronal damage signs (infarcts and pyknotic nuclei) in a manner similar to the positive control but the infarcts were smaller (Fig. 7e, f). Treatment with TAN-NLCs exhibited normal neuronal histological findings but massive bleeding from dilated blood vessels was still evident (Fig. 7g, h). On the other hand, CS-TAN-NLCs accomplished lack of any necrotic symptoms together with restoration of the normal histoarchitecture of many neurons, highlighting its ability to prevent the spread of damage to the prefrontal cortex (Fig. 7i, j).

Histological examination results for both striatum and prefrontal cortex provided a strong proof for the surpassing neuroprotective and anti-depressant potential of the elaborated CS-TAN-NLCs.

#### Immunohistochemistry of tyrosine hydroxylase (TH)

Tyrosine hydroxylase (TH) is an important enzyme involved in catecholamine biosynthesis as dopamine (DA). Therefore, alteration in its expression or activity has a significant influence on DA production and thereby is of great importance in the pathogenesis of PD [63].

**Fig. 8:**
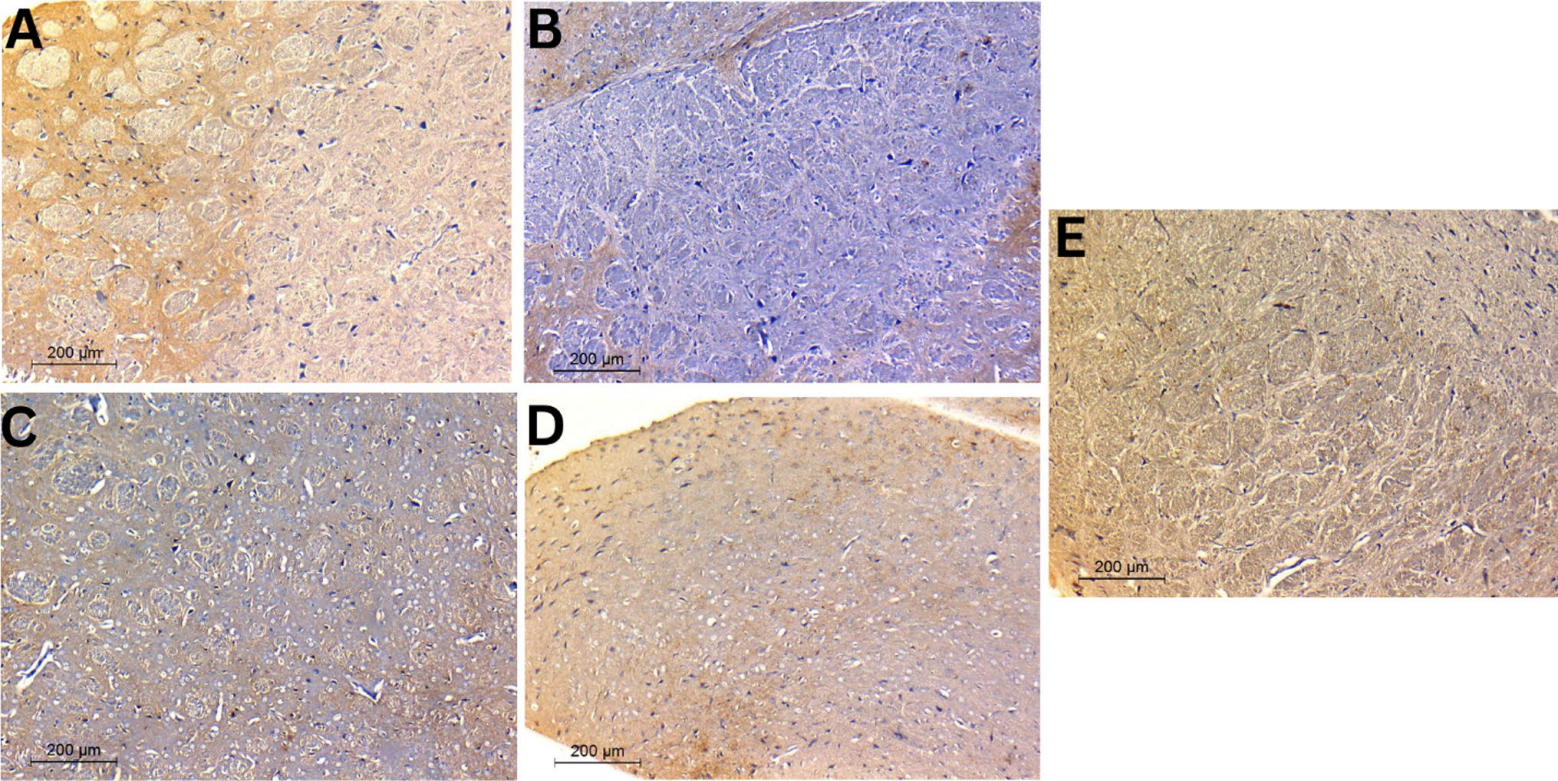
Photomicrographs of immunohistochemical staining of tyrosine hydroxylase in striatal neurons of different treated rats; **A** negative control, **B** positive control, **C** TAN-suspension, **D** TAN-NLCs, and **E** CS-TAN-NLCs. Magnification is × 200; the scale bar represents 100 μm

To investigate integrity of the nigrostriatal pathway, TH immunohistochemistry was carried out on striatal sections of different study groups and results are shown in Fig. 8. Herein, the primary antibody anti-TH was used for immunohistochemical staining to detect DA neurons as reported previously [64]. Immunolabeling using peroxidase-DAB system gave rise to brown precipitation in the cell body, axon, and dendrite process in TH-immunoreactive cells [64]. Differences in TH protein expression were indicated by differences in the intensity of TH-immunoreactive cells in immunohistochemical staining (reflected by brown color). As shown in Fig. 8, immunohistochemical TH staining in the striata of tested rats illustrated consistent results with those obtained from behavioral, biochemical, and histopathological evaluation. Striatal sections of the negative control group exhibited the maximum intensity of TH-immunoreactive cells, verified by intense brown color, indicating high TH protein levels in dopaminergic neurons (Fig. 8A). This was attributed to the ability of health neurons to utilize TH in the conversion of tyrosine to L-Dopa and finally dopamine [64]. However, rotenone-treated rats displayed the least TH levels, indicated by minimum intensity of TH-immunoreactive cells (Fig. 8B). This reflected decreased utilization of TH enzyme due to the marked loss of dopaminergic neurons projected to the striatum and hence disruption of the motor circuit of the basal ganglia [64]. In contrast, different treatments showed different levels of TH in the following order: TAN suspension < TAN-NLCs < CS-TAN-NLCs (Fig. 8C–E). Again, CS-TAN-NLCs succeeded to nearly reverse the induced neuronal damage indicated by having similar intensity of TH-immunoreactive cells to the negative control group (Fig. 8E).

Conclusively, combined results of biochemical and histopathological evaluation verified the superiority of CS-TAN-NLCs in the treatment of induced PD versus both TAN-suspension and uncoated formulation thanks to achieving maximum brain accumulation along with the synergistic antioxidant and neuroprotective effect of chitosan.

## Conclusion

In the current work, TAN-NLC formulations (TAN-NLCs and CS-TAN-NLCs) were employed to increase brain bioavailability of the anti-inflammatory neuroprotective phytomedicine (TAN) via a noninvasive intranasal route in rotenone induced PD rat model. As far as we are aware, investigation of the antiparkinsonian effect of the developed formulations in treatment of PD was performed for the first time. Additionally, the antidepressant action of TAN was explored via behavioral and histopathological examination of prefrontal cortex sections from treated animals. Furthermore, cathepsin B as a new anti-inflammatory therapeutic target for TAN was evaluated. Both intranasal TAN-NLCs and CS-TAN-NLCs significantly enhanced the therapeutic efficacy of TAN in PD. This was verified by various behavioral, biochemical, and histological evaluations compared to free TAN-suspension with superior effects achieved by the coated formulation (CS-TAN-NLCs). Consequently, CS-TAN-NLCs offer a highly adaptable strategy for effective intranasal brain delivery of TAN in treatment of PD.

## Data Availability

The authors confirm that the data for this study finding are available within the article.

## Declarations

### Animal studies

All institutional and national guidelines for the care and use of laboratory animals were followed.
